# The human G93A-SOD1 mutation in a pre-symptomatic rat model of amyotrophic lateral sclerosis increases the vulnerability to a mild spinal cord compression

**DOI:** 10.1186/1471-2164-11-633

**Published:** 2010-11-15

**Authors:** Natasa Jokic, Ping K Yip, Adina Michael-Titus, John V Priestley, Andrea Malaspina

**Affiliations:** 1 Centre for Neuroscience and Trauma, Blizard Institute of Cell and Molecular Science, Barts and The London School of Medicine and Dentistry, Queen Mary University of London

## Abstract

**Background:**

Traumatic injuries can undermine neurological functions and act as risk factors for the development of irreversible and fatal neurodegenerative disorders like amyotrophic lateral sclerosis (ALS). In this study, we have investigated how a mutation of the superoxide dismutase 1 (SOD1) gene, linked to the development of ALS, modifies the acute response to a gentle mechanical compression of the spinal cord. In a 7-day post-injury time period, we have performed a comparative ontological analysis of the gene expression profiles of injured spinal cords obtained from pre-symptomatic rats over-expressing the G93A-SOD1 gene mutation and from wild type (WT) littermates.

**Results:**

The steady post-injury functional recovery observed in WT rats was accompanied by the early activation at the epicenter of injury of several growth-promoting signals and by the down-regulation of intermediate neurofilaments and of genes involved in the regulation of ion currents at the 7 day post-injury time point. The poor functional recovery observed in G93A-SOD1 transgenic animals was accompanied by the induction of fewer pro-survival signals, by an early activation of inflammatory markers, of several pro-apoptotic genes involved in cytochrome-C release and by the persistent up-regulation of the heavy neurofilament subunits and of genes involved in membrane excitability. These molecular changes occurred along with a pronounced atrophy of spinal cord motor neurones in the G93A-SOD1 rats compared to WT littermates after compression injury.

**Conclusions:**

In an experimental paradigm of mild mechanical trauma which causes no major tissue damage, the G93A-SOD1 gene mutation alters the balance between pro-apoptotic and pro-survival molecular signals in the spinal cord tissue from the pre-symptomatic rat, leading to a premature activation of molecular pathways implicated in the natural development of ALS.

## Background

Mutations of the superoxide dismutase 1 (SOD1) gene cause degeneration primarily at the level of the spinal cord motor neurone pool and this detrimental effect may be accelerated by environmental stressors. SOD1 gene mutations have been found in approximately 20% of individuals with the inherited form of amyotrophic lateral sclerosis (ALS) motor neuron disease (MND), a rapidly progressive and fatal neurological disorder [1]. Neurodegeneration in ALS may result from genetic factors predisposing motor and glial cells to a higher level of vulnerability to different types of injuries, like mechanical stress [2-5]. Retrospective studies have shown how spondylotic myelopathy, a history of bone fracture and of surgical treatment are significantly more represented among ALS individuals compared to the general population [3]. Likewise, ALS appears to be 5 to 6 folds more prevalent in Italian professional footballers [3,5]. If mechanical stress plays a part in the unravelling of ALS as suggested by these epidemiological observations, it would be important to understand how the cascade of stress-induced molecular events precipitates or simply accelerates the development of ALS in genetically susceptible individuals.

We have recently shown that a mild compression spinal cord injury (SCI) and the over-expression of the G93A-SOD1 gene in the rat can induce the time-dependent transcriptional regulation of the same molecular responses modulating oxidative stress, apoptosis, inflammation, membrane ion transport and the neurofilaments homeostasis [6]. These molecular pathways have already been extensively investigated in these animal models of spinal cord degeneration [7-14]. The detrimental effect of neurofilaments aggregation on axonal transport is widely acknowledged [13], whilst the identification of motif deletions of the heavy neurofilament subunit gene [(Nfh); [15]] and of an increase in the levels of Nfh in the cerebrospinal fluid from ALS patients support the hypothesis that these cytoskeletal proteins are central in the pathogenesis of ALS [15,16].

High-penetrance genetic defects like mutations of the SOD1 gene may not only lead to a neurodegenerative disorder but could also increase the nervous tissue susceptibility to trauma in a pre-symptomatic stage. G93A-SOD1 toxicity has already been shown to increase the vulnerability of motor neurons and muscles to sciatic nerve injury, by reducing the post-injury motor unit survival whilst impairing the muscle contractile characteristics [17].

In this study, we have investigated by large-scale gene expression analysis how the G93A-SOD1 gene mutation modifies the acute molecular response to a mild compression injury in a pre-symptomatic rat. The low-intensity mechanical stress employed in this study causes no major tissue damage and healthy rats normally undergo full locomotor recovery within a short-time from injury [18,19]. We have also evaluated the post-injury expression profile of Nfh in the G93A-SOD1 transgenic rats and in their wild type (WT) littermates, together with the determination of spare white matter, of macrophage infiltration, of microglial activation and of the number and size of motor cells in the spinal cord segment immediately caudal to the site of injury. In the same one-week post-injury time-frame, we have compared the recovery of locomotor functions of the G93A-SOD1 rats to the one observed in WT rats after compression SCI. This study provides further insight into the toxic effects induced by a SOD1 gene mutation, showing how the molecular response to a mechanical trauma can be profoundly modified by this genetic defect in an otherwise pre-symptomatic rat.

## Results

### The G93A-SOD1 rats display a poor locomotor recovery in the first week following mild spinal cord compression

**Figure 1:**
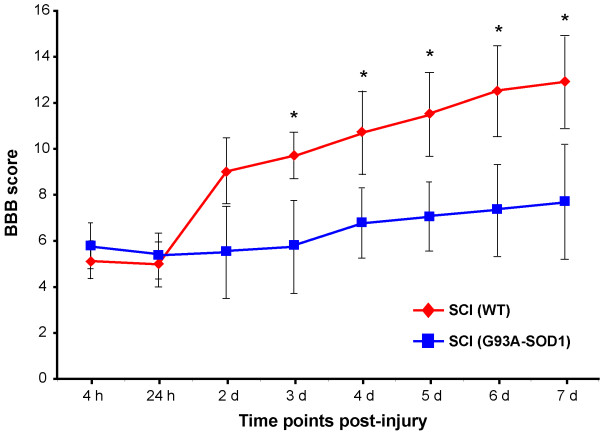
**Temporal pattern of recovery of locomotor functions after mild compression SCI in the G93A-SOD1 rats and in the WT littermates**. WT animals show a marked functional recovery between 1 and 2 days, followed by a steady locomotor improvement for the remaining period of observation. WT rats display an improved motor function compared to G93A-SOD1 rats from day 2 to day 7 of the post-injury experimental period (the difference between the WT and G93A-SOD1 locomotor functions is statistically significant only from the day 3, * P < 0.05), whereas G93A-SOD1 rats only appeared to perform slightly better, but non-significantly than the WT littermates at 4 hours and at 1 day post-injury. BBB scoring prior to injury of the G93A-SOD1 and the WT rats showed no locomotor dysfunction and no differences in BBB functional scores between the two genetic types (BBB score >21; normal locomotor functions). Error bars represent SEM. N: 5 animals per group were used.

We have previously reported the significant locomotor improvement that WT animals undergo in a 7 day time-period following compression spinal cord injury (SCI) [6]. Prior to surgery, the mean baseline locomotor performances measured using the BBB open-field locomotor rating scale as previously reported [20], were found not to be significantly different between the G93A-SOD1 and the WT groups [both above 21; normal presence of movements]. However, comparative BBB scoring of WT and G93A-SOD1 rats in the 1-week observation period following compression SCI showed significant differences (Figure 1). The G93A-SOD1 animals appear slightly less impaired at 4 hours and at 1 day from compression SCI than their WT littermates, although this difference was not statistically significant. The G93A-SOD1 animals show only a marginal improvement after compression SCI throughout the period of observation, whilst WT rats display a locomotor recovery between the first and the second day after compression SCI, followed by a much higher level of recovery throughout the 7 day period compared to the G93A-SOD1 rats (the difference in rated locomotor functions is statistically significant from the 3^rd ^day, P < 0.05; Figure 1). The BBB scale describes changes in the articulation of movements of the hip, knee, ankle, tail and posterior feet position. At the 7 day post-injury time period, the WT animals achieved a mean BBB locomotor rating score of 13 which is classified as "...constant weight support in the plantar step, constant hindlimbs and forelimbs coordination". At the 7 day post-injury time period, the G93A-SOD1 rats achieved a mean BBB locomotor rating score of 8, which is categorized as "... frequent or consistent weight support in the dorsal step and no support in plantar step, soft movements without supporting the body weight" [20]. The molecular basis of genetic vulnerability of G93A-SOD1 to trauma was further investigated.

### Comparative analysis of the gene expression changes in G93A-SOD1 and in wild type spinal cords following mild compression spinal cord injury

We have used an Illumina Bead-chip platform to compare the post-injury gene expression profile in spinal cord from G93A-SOD1 transgenic rats to the genetically homogeneous age-matched wild type (WT) littermates subjected to the same mild compression SCI procedure. We have pooled spinal cord samples containing the epicenters of injury obtained from WT or G93A-SOD1 transgenic rats sacrificed at different time points from the compression SCI (on average, 5 spinal cord samples in each pool; see Methods). We have focussed our studies on the first 7 days after injury, since post-traumatic cell loss by necrosis and apoptosis involving large ventral horn motor neurones and glial cells is at its highest levels in this time-window [9]. The data obtained from the gene expression analysis have been submitted to gene expression omnibus (GEO), and can be found in this repository with the series entry GSE22161 http://www.ncbi.nlm.nih.gov/geo/query/acc.cgi?acc=GSE22161. Supplementary files can be retrieved using appropriate links available from the same database series entry.

**Figure 2:**
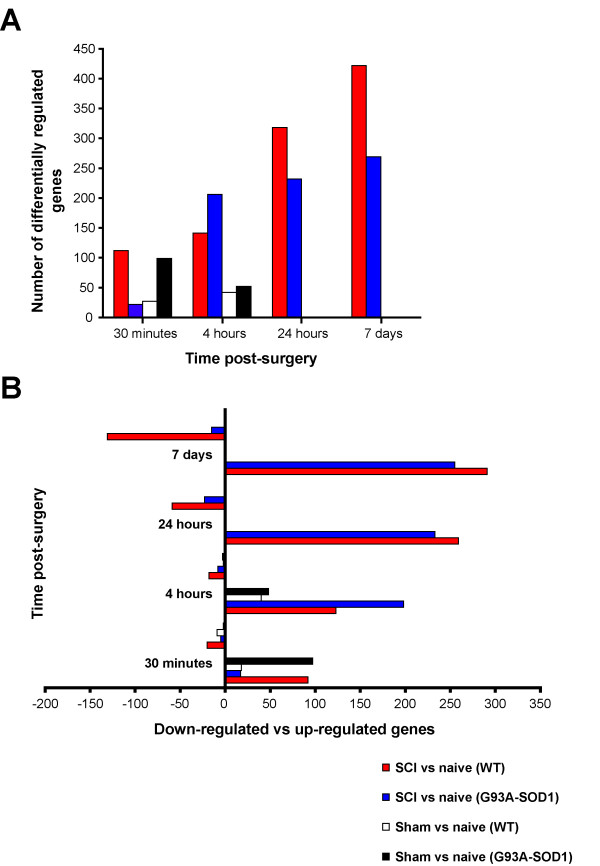
**Number of differentially regulated genes at the site of compression spinal cord injury in the G93A-SOD1 rats and in the WT littermates**. A. Graph displaying the total number of differentially regulated genes in the 10 week old WT and G93A-SOD1 spinal cord samples at the site of compression SCI. The largest difference in the number of differentially expressed gene candidates between the WT and the G93A-SOD1 spinal cord tissues is seen at the 7-day post-injury time point. B. Break-down of up-regulated versus down-regulated genes in the WT and G93A-SOD1 spinal cords at different time points from compression injury. The most remarkable difference between the two genetic types is the down-regulation of 135 genes in WT injured spinal cord tissue at 7 days post-injury, whilst G93A-SOD1 injured tissue down-regulate only 10 genes. Control: naïve and genetically-matched spinal cord samples (from 10 week old pre-symptomatic rats) were used as reference tissues in the differential gene expression analysis. Five animals per a group were used.

The number of differentially regulated genes in injured WT and G93A-SOD1 spinal cords compared to genetically-matched uninjured tissues is reported in Figure 2A, for each of the chosen post-injury time-point. At the 30 minutes, 24 hour and 7 days time points, the total number of differentially regulated genes after compression injury was higher in WT than in G93A-SOD1 spinal cord. Comparison of down-regulated versus up-regulated genes (Figure 2B) revealed that the increased number of differentially regulated genes in the WT spinal cord at the 7 day time-point compared to G93A-SOD1 spinal cord was due to down-regulated genes. The spinal cords from the G93A-SOD1 transgenic rats showed a larger number of up-regulated genes compared to WT spinal cords at the 4 hour post-injury time point. Figure 2A and 2B also show that sham surgery in G93A-SOD1 and WT animals induced the differential regulation of a number of gene candidates, particularly in G93A-SOD1 spinal cord at 30 minutes from compression SCI (when compared to genetically and age-matched naïve animals). The correlation analysis (BeadStudio-3 scatter plot) between the WT and the G93A-SOD1 spinal cord expression profiles after injury and genetically age-matched naïve tissues corresponds to the differences seen in the raw number of differentially regulated genes reported above. Whilst r^2 ^values for the correlation between SCI and naïve tissues are comparable for G93A-SOD1 and WT spinal cords at 4 hours and 24 hours after injury (4 hours WT, r^2^: 0.9414; 4 hours G93A-SOD1, r^2^: 0.9389; 24 hours WT, r^2^: 0.8588; 24 hours G93A-SOD1, r^2^: 0.8837), the correlation coefficients diverge significantly at the 7-day time point, with a r^2 ^of 0.889 for the G93A-SOD1 spinal cord and a r^2 ^of 0.809 for the WT spinal cord. A lower correlation coefficient indicates a higher level of differential regulation of the Bead-array 22,418 test-genes in the spinal cord samples under investigation. The r^2 ^value of the technical replica experiment performed probing two separate bead-arrays with the same RNA sample was 0.9876.

**Figure 3:**
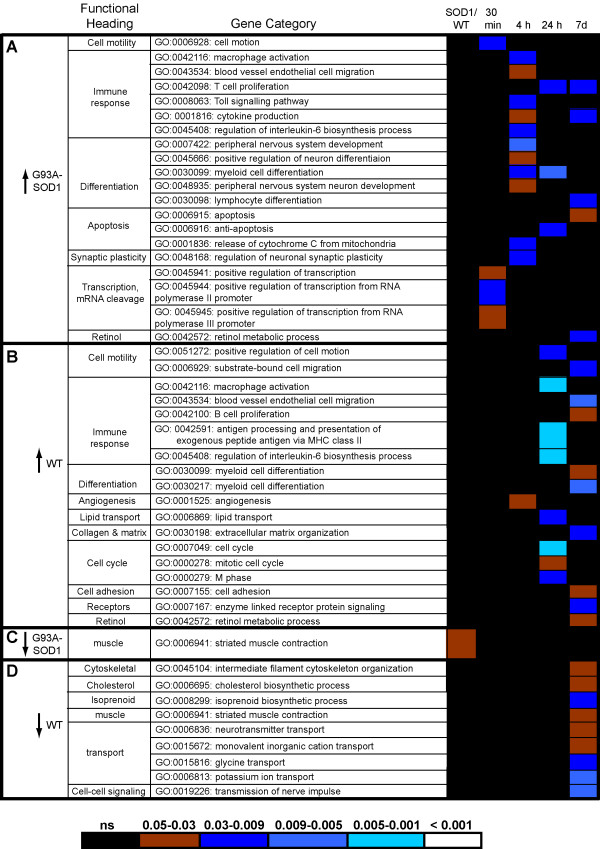
**Comparative gene expression analysis by *High-Throughput GoMiner *of spinal cord samples from G93A-SOD1 and WT rats subjected to a mild compression SCI**. The heat chart displays the Gene ontology (GO) categories (grouped into main functional headings) computed by *High Throughput GoMiner *computational analysis of the differentially expressed genes identified in spinal cord from WT and G93A-SOD1 rats after compression SCI, using naive (10 week old) spinal cord tissue from rats of the same genetic type as reference. *High Throughput GoMiner *defines the biological significance of the gene expression changes according to a multilayered process of statistical processing. GO categories are selected on the basis of their high level of enrichment of the differentially expressed genes, using a false discovery rate correction (FDR) cut off of <0.05. FDR introduces a multiple comparisons correction, allowing the exclusion of those gene categories that would appear enriched simply by chance. Functionally similar GO categories are grouped within the same heading and reported as up-regulated predominantly in G93A-SOD1 spinal cord (A, ↑G93A-SOD1), in WT spinal cord (B. ↑WT), down-regulated in G93A-SOD1 spinal cord (C. ↓G93A-SOD1) and in WT spinal cord (D. ↓WT). The various levels of significance of differential regulation for each gene category are represented in the heat-chart with different colour codes (the correlation between colour codes and FDR values is reported at the bottom of the heat-chart). G93A-SOD1/WT column: Comparison between the gene expression profiles of spinal cord samples from G93A-SOD1 and WT naïve rats (10 week of age).

We have used *High Throughput GO-Miner *as previously reported [6,21] to obtain an integrated ontological analysis of the gene expression datasets of injured G93A-SOD1 and WT spinal cords. This program identifies gene categories within the Gene Ontology (GO) database that are enriched with the differentially regulated genes under investigation and display a significant false discovery rate (FDR < 0.05), a value which indicates the statistical significance of the identified gene category by eliminating probe signals detected only by chance [21]. Of the 150 gene categories computed by *High Throughput GO-Miner*, 36 with a broad biological significance and not related to any specific functional or biological mechanism (e.g. GO:0007610_behavior; GO:0019725_cell_homeostasis; GO:0009893_positive_regulation_of_metabolic_process; GO:0042592_homeostatic_process) were excluded from further analysis, as were 66 gene categories showing the same temporal distribution and level of FDR significance in G93A-SOD1 and in WT spinal cord. The time dependent expression of the remaining 48 gene categories has been graphically displayed in a heat-chart, where the FDR value for each gene category at a specific time point is represented with a colour-code (Figure 3). The selected gene categories are grouped into functional headings, according to their "parent-child" relationship and biological affinity. Gene categories found to be up-regulated in G93A-SOD1 spinal cord, those up-regulated in WT spinal cord, those down-regulated in G93A-SOD1 and those down-regulated in WT spinal cord are reported in Figure 3A, B, C and 3D respectively. Those gene categories up-regulated predominantly in G93A-SOD1 spinal cord seem to be mostly detected at 4 hours post-injury (Figure 2A), whereas those differentially regulated in WT spinal cord are identifiable mainly at the 24 hour and 7 day time points (Figure 3B, D). The key changes shown in Figure 3 will now be briefly reviewed:

#### The 30 min time point

Only four gene categories are differentially regulated at the 30 minutes post-injury time point, all of which are up-regulated in the G93A-SOD1 spinal cord and mainly involved in transcriptional mechanisms and in cell motility.

#### The 4 hour time point

Among those gene categories activated only in G93A-SOD1 spinal cord at this time point, some supervise the development of neuronal and myeloid cells, the differentiation of lymphoid organs and the regulation of the synaptic plasticity (Figure 3A). At this time point, gene candidates with a pro-apoptotic effect and involved in the release of cytochrome C from mitochondria, become significantly up-regulated in G93A-SOD1 spinal cord, together with other genes involved in macrophage migration, in the interleukin-6 and TNF-alpha molecular cascades and in the regulation of T-cell proliferation and cytokine production. The only gene category selectively regulated in WT spinal cord at the 4 hour time-point contains gene candidates involved in angiogenesis (GO:0001525; Figure 3B).

#### The 24 hour time point

As previously reported [6], WT spinal cord presents, at this post-injury time point, gene expression changes with a likely pro-survival effect, such as the up-regulation of genes involved in lipid transport (Figure 3B). Other molecular responses that may be detrimental to spinal cord functional recovery are also prominent, including the activation of macrophage and interleukin-6 molecular pathways (Figure 3B). Unlike G93A-SOD1 spinal cord tissue, WT spinal cord up-regulates gene candidates involved in cell cycle control as previously reported in a rat model of mild spinal cord injury [8,22].

#### The 7 day time point

This time-point is characterised by further up-regulation of pro-survival signals in WT spinal cord, including gene candidates involved in cell adhesion and in the formation of the extra-cellular matrix. As reported above, the 7-day time-point after compression SCI displays the most remarkable difference between G93A-SOD1 and WT spinal cord in the post-injury period in study (Figure 2A, B). This is represented by the down-regulation only in WT spinal cord of 135 genes (Figure 2B), which are part of 9 gene categories according to our *High Throughput GO-Miner *analysis (Figure 3D). These down-regulated genes supervise the homeostasis of intermediate neurofilaments, cholesterol and isoprenoid metabolism, striated muscle contraction, ion and neurotransmitter transport, and cell-cell signalling.

**Figure 4:**
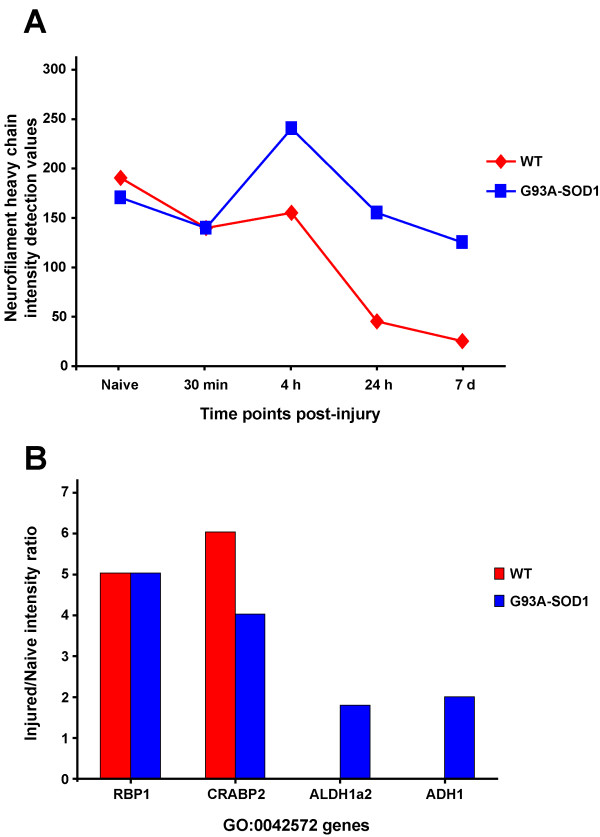
**Differential regulation in injured spinal cord tissue from WT and G93A-SOD1 rats of the intermediate neurofilament heavy chain and of genes involved in retinol metabolism according to Bead-array analysis**. **A**. Profile of post-injury differential expression of the neurofilament heavy chain (Nfh) in the WT and in the G93A-SOD1 spinal cords (intermediate neurofilaments gene category, GO:0045104), according to the Bead-array gene expression analysis. Note the higher level of expression of the neurofilament in G93A-SOD1 spinal cord at all the post-injury time points and its significant level of down-regulation in WT spinal cord at 7 days from injury compared to naive tissue. **B**. The graph reports the 7 day post-injury up-regulation in WT and G93A-SOD1 injured spinal cord of genes included in the retinol metabolism gene category (GO:0042572), including retinol binding protein 1 (RBP1) and cellular retinoic acid binding protein 2 (CRABP2). The up-regulation is expressed as the ratio of intensities between injured and naive tissues. Two additional genes within the GO:0042572 gene category, the alcohol dehydrogenase 1 (ADH1) and aldehyde dehydrogenase 1, member A2 (ALDH1a2) are up-regulated only in the G93A-SOD1 spinal cord at 7 days from injury.

More detailed analysis of the Bead-array results indicates that the genes included in the intermediate neurofilaments GO category (GO:0045104), and particularly the neurofilament heavy chain (Nfh), show a significant drop in expression in WT spinal cord at 7 days from injury, whilst its expression transiently increases between 4 and 24 hours from compression SCI (Figure 3D; 4A). G93A-SOD1 spinal cord shows a similar pattern of temporal expression change for Nfh, but this intermediate neurofilament maintains a higher level of expression compared to WT spinal cord and no significant drop in expression between 24 hours and 7 days (Figure 4A; FDR < 0.05). The SCI-induced differential regulation of Nfh has been further characterised by real-time RT-PCR and immunohistochemistry as reported below. Another relevant feature of the post-injury spinal cord molecular profile at the 7-day time-point is the up-regulation of a gene category involved in retinoid metabolism (GO:0042572; Figure 3A, B). This gene category contains 34 genes which control retinol metabolism and the homeostasis of vitamin A, 12 of which are reported in the Bead-array utilized in this study. The post-injury up-regulation of GO:0042572 is shown in both spinal cord genetic types in this study, (Figure 3A, B) at 7 days post-injury, with a higher level of statistical significance for the G93A-SOD1 spinal cord (FDR:0.003) compared to WT spinal cord (FDR:0.04). Our Bead-array analysis shows that retinol binding protein 1 (RBP1) and cellular retinoic acid binding protein 2 (CRABP2) display a significant level of up-regulation at 7 days compared to naïve and genetically matched spinal cord tissue in both WT and G93A-SOD1 spinal cord, whilst only G93A-SOD1 spinal cord shows up-regulation of Alcohol dehydrogenase 1 (ADH1) and of Aldehyde dehydrogenase 1, member A2 (ALDH1a2) (Figure 4B).

#### Gene expression changes after laminectomy

**Figure 5:**
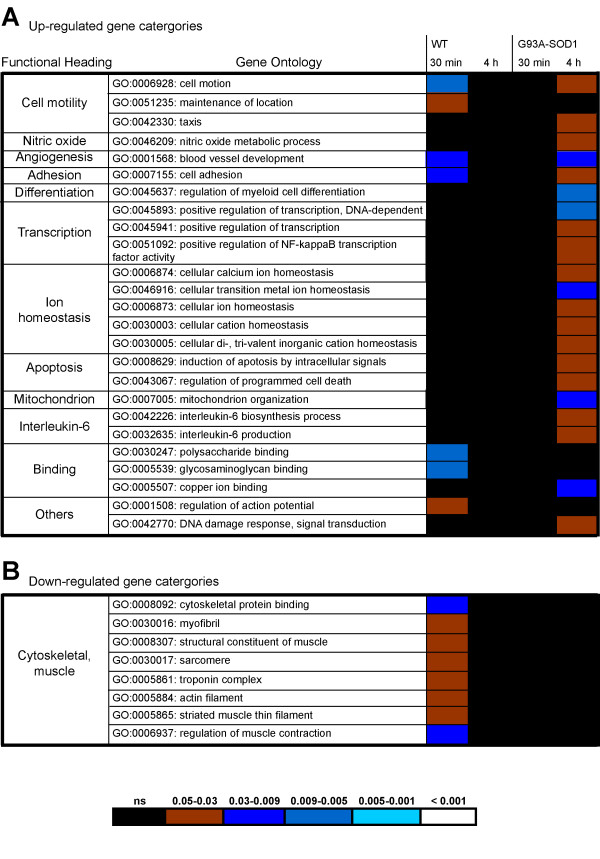
***High throughput GoMiner *ontological analysis of the differentially regulated genes in WT and G93A-SOD1 spinal cord at 30 minutes and 4 hours after laminectomy**. The heat chart displays headings containing clusters of functionally related Gene ontology (GO) categories, computed using *High Throughput GoMiner*. Those gene categories found to be up-regulated in WT and G93A-SOD1 spinal cord tissue after laminectomy compared to naive and genetically matched spinal cord tissues are displayed in Figure 5A, whilst the gene categories which become down-regulated are reported in Figure 5B. At 4 hours from laminectomy, a large number of gene categories are over-expressed only in G93A-SOD1 spinal cord. At the 30 minutes time point, WT spinal cord gene expression differs from G93A-SOD1 tissue for the down-regulation of a number of cytoskeletal gene categories. 10 week old genetically age-matched spinal cord tissues were used as reference. Other: categories which include genes involved in the regulation of the action potential, of the DNA damage response and of signal transduction.

Our gene expression study indicates that sham-operation induces the differential regulation of a smaller number of genes in the spinal cord at both the 30 minutes and at the 4 hours post-injury time points, when compared to the amount of gene expression changes generated by mild compression SCI (Figure 2A, B). *High Throughput Go-Miner *computation of the gene expression changes induced by laminectomy in these spinal cord tissues at the 30 minutes and at the 4 hours post-injury time points has disclosed a total of 126 gene categories. Like for the compression SCI experiment, 33 general gene categories were excluded from further analysis, whilst 60 gene categories appeared to have the same pattern of expression and level of significance in G93A-SOD1 and in WT spinal cord. Figure 5 shows the remaining 33 gene categories with a FDR < 0.05 in either G93A-SOD1 or WT spinal cord, at 30 minutes and 4 hours from laminectomy. Most of the differentially expressed gene categories are up-regulated in the G93A-SOD1 spinal cord, 4 hours after laminectomy. The genes included in these categories encompass a wide range of functional processes including nitric oxide metabolism, cell adhesion, ion homeostasis, transcription, apoptosis, mitochondrion organization and biogenesis, and interleukin-6 biosynthesis (Figure 5A). Eight gene categories containing a wide range of genes related to the muscle cytoskeletal structure (e.g. myofibril, sarcomere, troponin complex, actin filaments, muscle contraction machinery) are significantly down-regulated only in WT spinal cord 30 minutes after laminectomy (Figure 5).

#### Direct comparison of G93A-SOD1 vs WT spinal cord tissue

**Figure 6:**
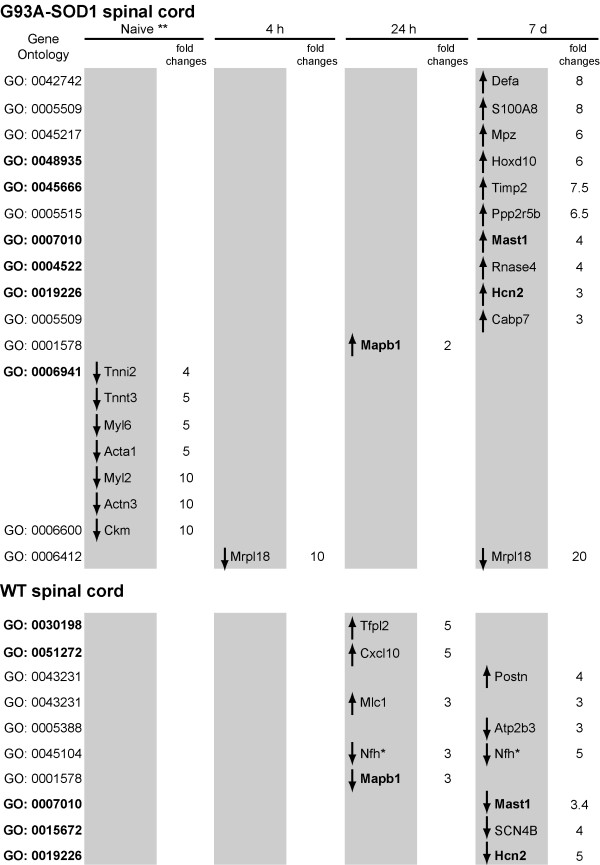
**Gene candidates showing a significant differential regulation comparing the G93A-SOD1 to the WT spinal cord after mild compression SCI**. 31 gene candidates have been found to have a significant level of differential expression comparing G93A-SOD1 and WT naïve spinal cord tissues at 10 weeks of age (*) and the same tissues at different time points after mild compression SCI (Bead-array analysis). Each gene's differential regulation is further characterised by comparing the injured spinal cord tissue with genetically-matched naive spinal cord tissue and reported as fold changes of the ratio injured/naïve tissues. The genes are sub-divided according to whether their expression change occurs in WT spinal cord, in G93A-SOD1 spinal cord or in both (genes reported in bold) and to whether their expression increases (↑) or decreases (↓) compared to naïve genetically-matched spinal cord tissues. The majority of genes showing differential expression after SCI are identified in the 24 hours and in the 7 days time points. Mapb1, Hcn2 and Mast1 (reported in bold) are differentially regulated in both WT and G93A-SOD1 spinal cords and the differential regulation seems to go in the opposite way for each gene candidate in the two tissues in study when genetically age-matched naive tissue is used as reference. The GO categories which include the differentially regulated genes are also reported (in bold those already identified by GoMiner ontological analysis, Figure 2). *: Nfh expression appears to decrease significantly in the WT spinal cord at 24 hours and 7 days from compression SCI, compared to age-matched and genetically-matched naïve spinal cord.

Using BeadStudio-3, we have been able to directly compare the expression profiles of G93A-SOD1 and WT spinal cord tissues harvested at the selected time-points after compression SCI. We have identified 33 gene candidates with a significant level of differential regulation. Further evaluation of the nature of the differential regulation of these gene candidates in each tissue type was performed using genetically and age-matched naïve tissue as reference. The differentially regulated genes are reported in Figure 6, along with their reference GO categories. In each tissue type, genes are reported as up-regulated or down-regulated (compared to genetically-matched naïve tissue). In bold are those genes whose expression changes in both the WT and the G93A-SOD1 spinal cord, and those gene categories that have been selected by the *High Throughput GO-Miner *analysis (reported in Figure 3) and that contain these differentially regulated genes.

At 7 days after injury, the hyperpolarization-activated cyclic nucleotide-gated potassium channel 2 (Hcn2) appears to have one of the highest levels of differential regulation when directly comparing G93A-SOD1 to WT injured tissues. This is the result of its up-regulation in G93A-SOD1 spinal cord and of its remarkable down-regulation in WT spinal cord (Figure 6). Hcn2 is represented in a number of "parent-child" gene categories according to the GO database, which contain functionally related gene candidates involved in cell-cell signalling (GO:0019226) and ion transport (GO: 0006813). Other genes involved in membrane ion exchange and synaptic transmission show the same temporal pattern of down-regulation in WT spinal cord (though remaining unchanged in G93A-SOD1 spinal cord), including the voltage-gated sodium channel type IV beta subunit (SCN4B) and the ATPase Ca(2+) transporting plasma membrane 3 (Atp2b3; Figure 6). The same pattern of over-expression in G93A-SOD1 spinal cord and of down-regulation in WT spinal cord has been identified for the microtubule-associated protein B 1 (Mapb1) at 24 hours and for the microtubule-associated serine/threonine kinase family 1 (Mast1) at 7 days from compression SCI (Figure 6). Mapb1 has been reported to bind to microtubules promoting their stabilization within axons, and to facilitate the interplay between the microtubule system and microfilaments. Microtubules play a dominant role in the central growth cone and are essential for the maintenance and the elongation of the axon. Mast1 becomes up-regulated in the G93A-SOD1 spinal cord at 7 days from injury, whilst its expression diminishes in WT tissue at the same post-injury time point. MAST1 is part of the cell's scaffolding kinase activity, with a selective expression in oligodendrocytes and in white matter containing brain regions. Among cytoskeletal gene candidates that behave differently in the two genetic types of injured spinal cord, we have identified the myelin protein zero (mpz, P0) and Nfh. Mpz, an important constituent of the myelin sheet, undergoes a significant up-regulation in injured G93A-SOD1 tissue a 7 days from compression SCI, whilst the differential regulation of Nfh (GO:0045104) at 24 hours and at 7 days post-injury, is the result of a significant down-regulation only in WT spinal cord (Figures 3D, 6).

Among those genes undergoing a selective up-regulation in the G93A-SOD1 spinal cord and remaining unchanged in the WT tissue (Figure 6), we have identified alpha 1 defensin (Defa) and the tissue inhibitor of metalloproteinase 2 (Timp2). Defa is known to have antimicrobial properties and to be localised in the granules of neutrophils from where it can be secreted. Timp2 belongs to a group of matrix inhibitors of metalloproteinases, whose potential tissue repair-promoting effect has been recently exploited as a therapeutic target in acute brain injuries [23]. Furthermore S100A8 and calcium binding protein 7 (Capb7), both up-regulated in the G93A-SOD1 spinal cord, are predominantly expressed in glial cells.

As previously reported [6], among those gene candidates down-regulated in the G93A-SOD1 uninjured spinal cord (10 weeks of age), we have identified constituents of collagen, components of the muscle contractile machinery like the human troponin I fast-twitch isoform 2 and 3 (Tnni2/3) genes, the myosin light chain 2 and 6 (Myl2; Myl6), the **a**ctin - alpha skeletal muscle 1 (Acta1), the actinin -- alpha 3 (Actn3), and the creatine kinase -- muscle type (Ckm; GO: 0006941; Figures 3C, 6). The mitochondrial ribosomal protein L18 (Mrpl18) is also down-regulated in the G93A-SOD1 spinal cord at 4 hours and 7 days from compression SCI.

#### Real-time RT-PCR

**Figure 7:**
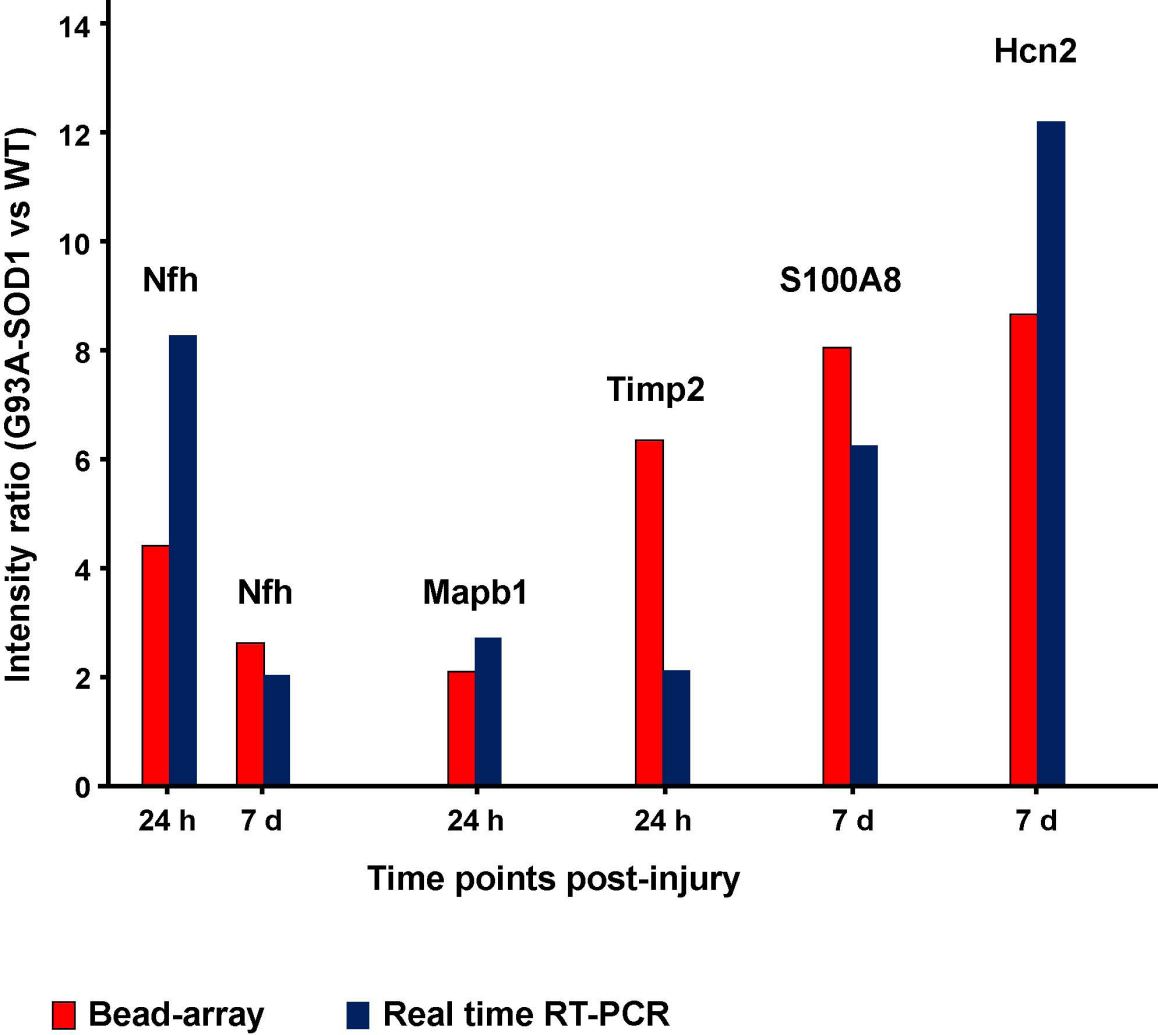
**Histogram representing the G93A-SOD1 versus WT ratios of the spinal cord intensity values detected for Nfh, Map1b, S100A8, Timp2 and Hcn2 at 24 hours and at 7 days from compression injury, as measured by Bead-array and real-time RT-PCR analysis**. The G93A-SOD1 vs WT expression ratios obtained using Bead-array (red code) and real time RT-PCR (blue code) analyses for Nfh, Map1b, S100A8, Timp2 and Hcn2 at two time points after compression SCI are reported. The two techniques of gene expression analysis confirm independently the same type of differential regulation for the selected gene candidates. S100A8 real-time RT-PCR confirms both the significant up-regulation in the G93A-SOD1 spinal cord detected by Bead-array analysis 7 days from compression SCI. Map1b RT-PCR has been included in order to validate the sensitivity of the real time RT-PCR and Bead array analyses for expression changes close to two-folds.

we have used real-time RT-PCR to confirm the differential regulation of Hcn2, Nfh, Timp2, S100A8 and Map1b, previously shown by Bead-array analysis. Figure 7 shows how both real-time RT-PCR and Bead-array analysis display a significant up-regulation in G93A-SOD1 spinal cord of the selected gene candidates, although the degree of differential regulation varies between the two techniques. Map1b differential regulation at 24 hours from injury, as demonstrated using real-time RT-PCR and Bead-array analysis, results from its up-regulation in G93A-SOD1 spinal cord and from its down-regulation in WT spinal cord (Figures 6, 7). The high G93A-SOD1 to WT Nfh expression ratio at 24 hours (and to a lesser extend at 7 days from injury) is the result of a significant down-regulation of this gene in WT injured spinal cord as already reported (Figures 6, 7).

### Expression of Nfh and of synaptophysin

**Figure 8:**
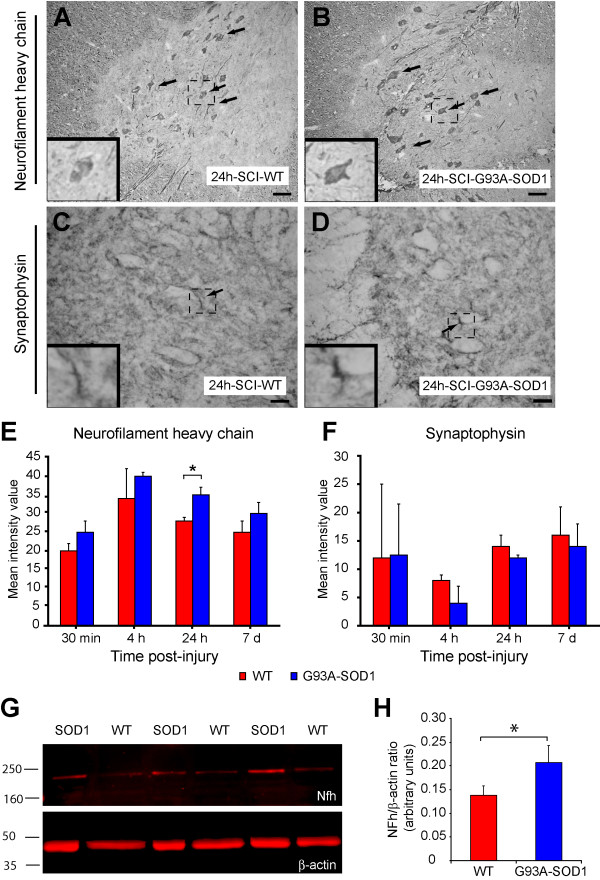
**Expression of neurofilament heavy chain and synaptophysin in ventral spinal cord at 24 hours after compression SCI**. Neurofilament heavy chain (Nfh; clone N52) staining of spinal cord sections is clearly visible in motor neurons (arrows) and adjacent axons in both WT (A) and G93A-SOD1 (B) which is significantly stronger in the G93A-SOD1 (* P = 0.03; E). Scale bar = 100 μm. Transverse sections are taken within a segment 10 mm caudal to the injury epicenter. Synaptophysin (SYN) immunoreactive synaptic boutons (arrows) can be seen surrounding unstained motor neuron cell bodies in both WT (C) and G93A-SOD1 (D) spinal cord, with no difference in SYN distribution between the two genetic types. The intensity of staining is non-significantly different between the G93A-SOD1 and wild type spinal cords (F). Scale bar = 25 μm. SCI-SOD1: spinal cord tissue from rats over-expressing the G93A-SOD1 gene mutation. SCI-WT: spinal cord tissue from WT rats. Western blot of spinal cord rostral to the injury epicenter obtained from WT and G93A-SOD1 rats. Three spinal cord samples for both WT and G93A-SOD1 were used (G). A significant increase in Nfh (clone 52) expression levels can be detected in G93A-SOD1 rats compared to WT rats with β-actin as the internal control (* P = 0.041; H).

To confirm that the spinal cord regulation of Nfh expression varies between WT and G93A-SOD1 animals at different time-points from injury (as shown using direct differential gene expression analysis by Bead-array and real-time RT-PCR, Figures 6, 7) we have carried out immunostaining for Nfh in comparable sections of spinal cord caudal to the epicenter of compression SCI, from WT and G93A-SOD1 rats (Figure 8A-B). Nfh expression by immunostaining appeared tendentially higher in the G93A-SOD1 spinal cord at all the post-injury time points considered when compared to the WT spinal cord, although only the 24 hour time point displayed a statistically significant difference (Figure 8E; p < 0.03). This significant difference in Nfh protein expression levels between the two groups at 24 h post-injury was further confirmed using Western blotting (Figure 8G-H).

We have also performed immunostaining of the similar sections for synaptophysin (SYN), a membrane glycoprotein of the pre-synaptic vesicles in neuron, whose expression does not seem to be altered following compression SCI according to our gene expression analysis (Figure 8C-D). Both Nfh and SYN have already been described to become differentially regulated in neurological structures subjected to mechanical injury [24,23]. SYN immunostaining shows selective staining of synaptic boutons, with no statistically significant difference between WT and G93A-SOD1 tissues of the protein expression of this gene at the post-injury time points considered (Figure 8F). In addition, the staining does not reveal any significant difference in SYN regional distribution in the G93A-SOD1 compared to the WT spinal cord. Both these proteins have been previously demonstrated to become differentially regulated in neurological structures subjected to mechanical injury [24,25].

### Histopathological assessment of the acute effects of mild compression spinal cord injury

**Figure 9:**
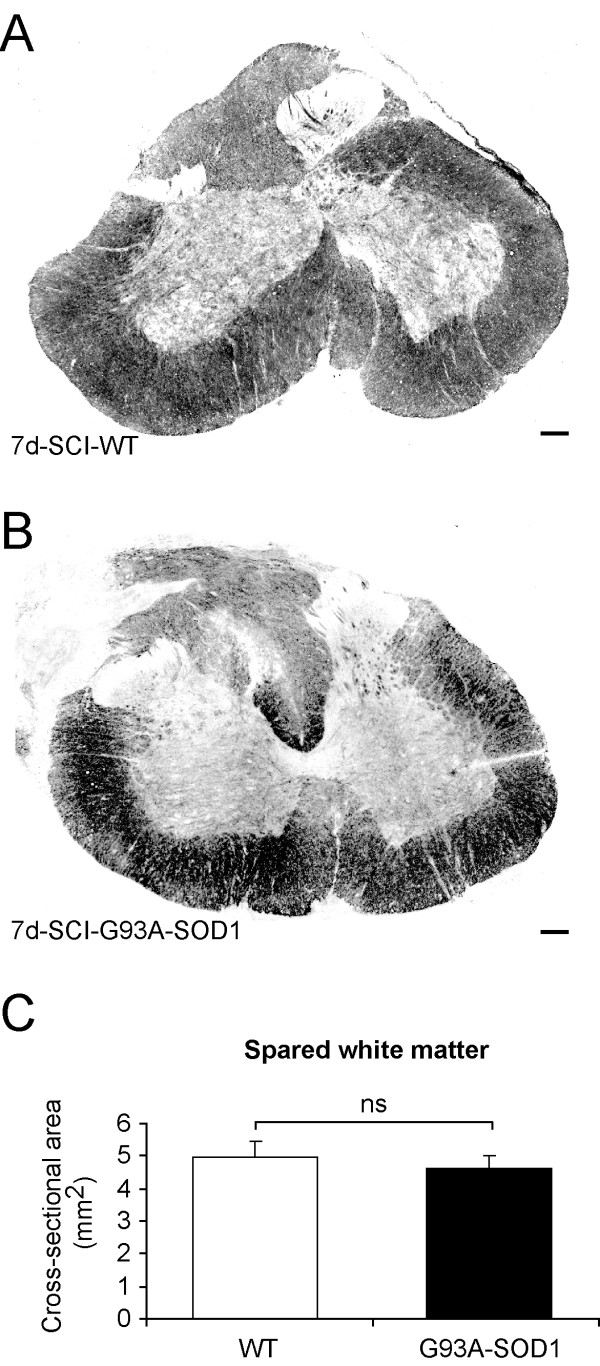
**Spared white matter in spinal cord caudal to compression epicenter**. Spared white matter stained with luxol fast blue can be seen clearly in WT (A) and G93A-SOD1 (B) in sections caudal to the compression epicenter 7 days after compression SCI. Morphometric analysis of the cross sectional areas of luxol fast blue stained white matter in WT and G93A-SOD1 showed no significant difference between groups (P = 0.30, C). ns = non-significant. Scale bar = 200 μm.

**Figure 10:**
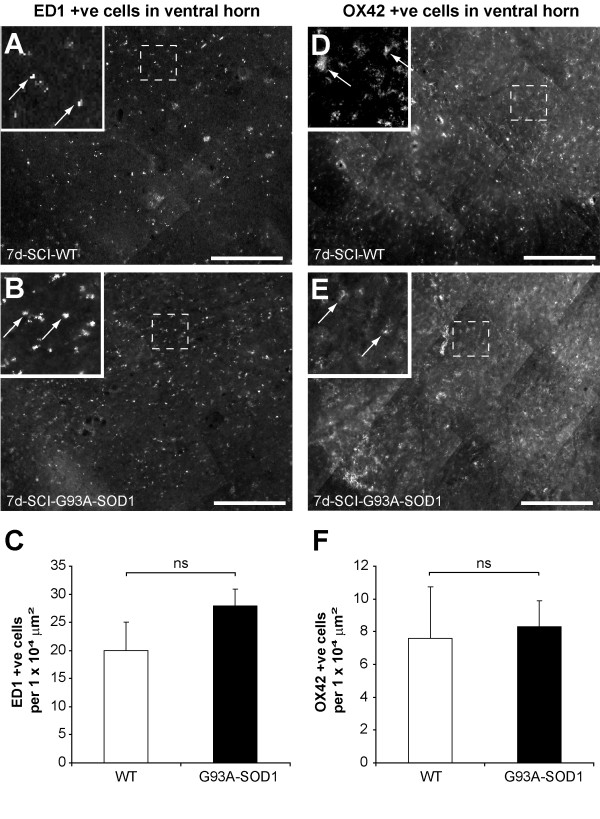
**Immunoresponse in spinal cord caudal to compression epicenter**. ED1 immunopositive cells (macrophages) were detected in the spinal cord ventral horn of WT (A) and G93A-SOD1 (B) in sections caudal to compression epicenter 7 days post-injury. Quantification showed an elevated but non-significant increase in ED1 immunopositive cells in G93A-SOD1 compared to WT spinal cord (P = 0.08, C). OX42 immunopositive cells (activated microglia) were detected in the spinal cord ventral horn of WT (D) and G93A-SOD1 (E) in sections caudal to compression epicenter 7 days post-injury. Quantification showed no significant difference in OX42 immunopositive cells in spinal cord of both groups (P = 0.76, F). ns: non-significant. Scale bar = 200 μm.

**Figure 11:**
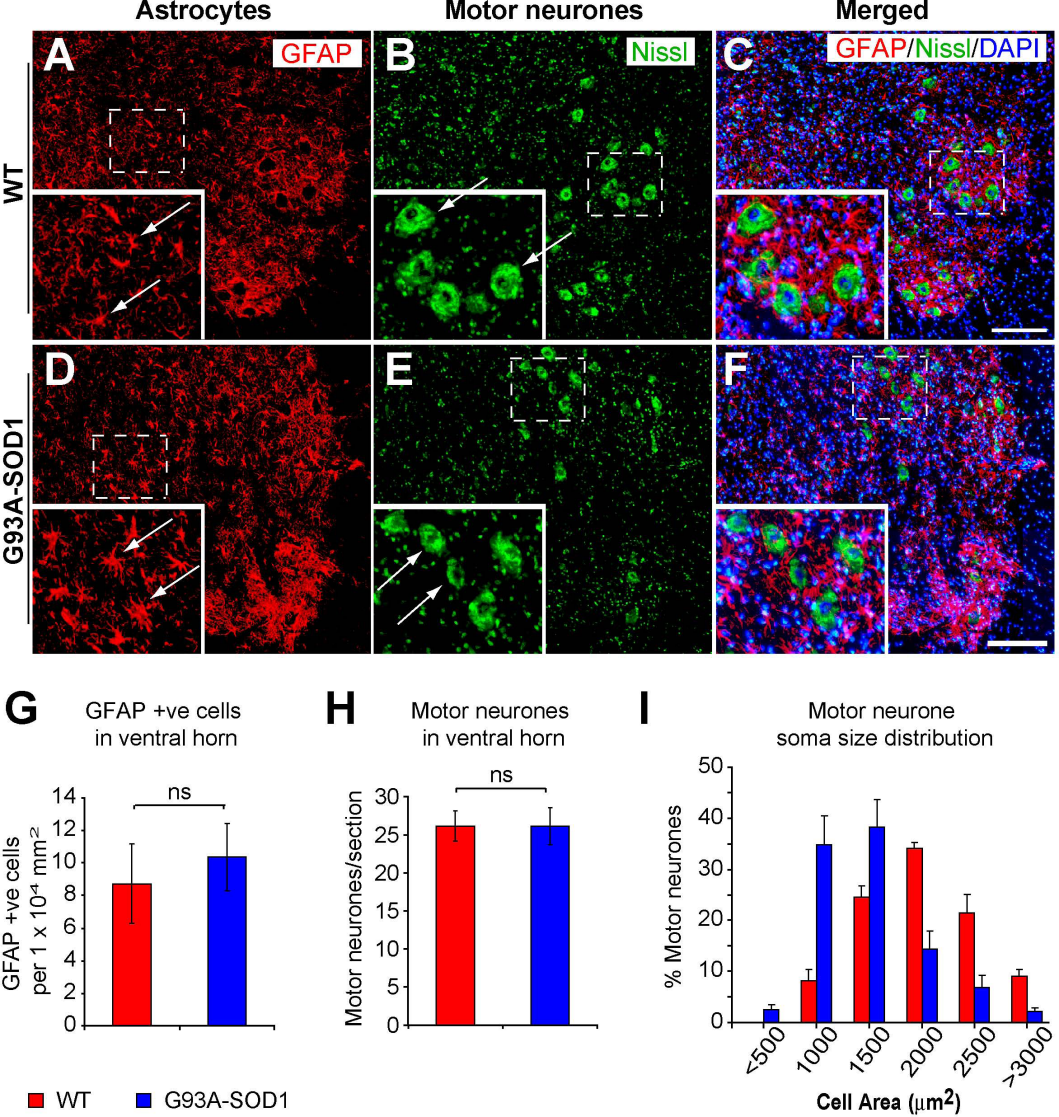
**Changes affecting astrocytes and motor neurones in spinal cord caudal to the compression epicentre**. GFAP immunopositive cells (astrocytes) were detected in the spinal cord ventral horns of the WT (A) and of the G93A-SOD1 (D) rats, in sections caudal to the compression epicenter 7 days post-injury. Quantification showed an elevated but non-significant increase in GFAP immunopositive cells in the G93A-SOD1 compared to the WT spinal cord (P = 0.31, G). Large Nissl stained cells (motor neurones) were detected in the spinal cord ventral horn of WT (B-C) and of G93A-SOD1 (E-F) in sections caudal to the compression epicenter 7 days post-injury. Although the number of motor neurones in the ventral horns are not significantly different between the two groups, further analysis of the motor cells soma size showed that the motor neurones in the WT spinal cord were larger than those identified in the G93A-SOD1 spinal cord (H-I). Kolmogorov-Smirnov test revealed a significant leftward shift in motor neurone soma size of the G93A-SOD1 spinal cord compared to WT spinal cord (P < 0.05, I). ns: non-significant. Scale bar = 200 μm.

We have used a luxol fast blue staining to examine spared spinal cord white matter following compression. The white matter appears to be mostly intact in the spinal cord segment caudal to the compression site (Figure 9). The comparison between the WT and the G93A-SOD1 rats showed that the area of spared white matter was not significantly different between the two groups (P = 0.30). Previous studies have identified increases in ED1 immunopositive macrophage infiltration, OX42 immunopositive activated microglia and GFAP immunopositive astrocytes in the spinal cord following injury [26,27]. At 7 days after spinal cord compression in the WT and in the G93A-SOD1 rats, there was an increase in the number of ED1-, OX42- and GFAP- immunopositive cells in the spinal cord caudal to the compression site (Figure 10-11. However, no significant difference was observed between the WT and the G93A-SOD1 rats (ED1, P = 0.08; OX42, P = 0.76; GFAP, P = 0.31).

### Reduced motor neurones size in the G93A-SOD1 spinal cord following compression spinal cord injury

To examine motor neurones, Nissl staining was used since the motor neurone marker choline acetyltransferase has been shown to be significantly reduced following spinal cord injury [28]. Motor neurone morphology can be clearly seen in the ventral horn of a spinal cord region caudal to the injury epicenter 7 days after mild compression SCI (Figure 11A-F). At this post-injury time point, the number of motor neurones in the ventral part of the spinal cord of WT rats did not differ from the number of motor neurones in the G93A-SOD1 rats (Figure 11H). However, motor neurones in the anterior horns from the G93A-SOD1 rats were significantly smaller in soma size (Kolmogorov-Smirnov test, p < 0.05) compared to those from WT littermates (Figure 11I).

## Discussion

This study shows how the over-expression of the G93A-SOD1 gene mutation modifies the molecular response to a mild mechanical compression in the spinal cord tissue from a pre-symptomatic rat. Using a histopathological assessment of the compression injury, we have found no significant differences between the WT and the G93A-SOD1 animals in the post-injury immunoresponse or in the amount of spared white matter following the trauma (Figures 10-11). Hence, despite delivering comparable spinal cord compression injuries to pre-symptomatic WT and G93A-SOD1 rats, G93A-SOD1 rats displayed a significantly lower level of post-injury locomotor recovery than their WT littermates. We observe that the significant differences in the post-injury locomotor recovery between the WT and the G93A-SOD1 rats are not due to differences in the induction of the injury, but rather to an intrinsically different molecular response to trauma in the two genetic variants of rats. A significantly low level of functional recovery has also been reported in G93A-SOD1 transgenic mice after sciatic nerve injury, with an acceleration of the disease progression so that 90 day old mice show deficits that are normally only seen at the end stage in uninjured G93A-SOD1 mice [17].

Whilst mutations of the SOD1 gene account only for approximately 2% of the whole ALS population, other not yet identified predisposing or causative genetic factors may act along the same lines, reducing the neuroregenerative potential and escalating the level of disability after trauma. These observations may partially explain the observed higher occurrence of ALS cases among individuals that have been exposed to repetitive mechanical trauma or that have been subjected to other forms of mechanical stress or surgical procedures [2,3,5].

For the purpose of our study, we have employed a well-characterised experimental platform of mechanical stress, which is known to cause only a mild damage to the spinal cord and to be associated with a good outcome in terms of post-traumatic locomotor recovery in rodents [29,19,30]. Other forms of injury cause a significant level of tissue destruction and a more florid profile of gene expression changes, masking subtle transcriptional changes that may be induced by the SOD1 gene mutation in an otherwise macroscopically healthy spinal cord. Under the experimental conditions of mild SCI, the G93A-SOD1 spinal cord displays a higher number of up-regulated genes compared to WT tissue at the 4 hour post-injury time point (Figure 2A-B). These include a broad range of genes involved in the modulation of macrophages, Toll-response, T and B-cell proliferation and in the production of interleukin-6 and TNF-alpha, featuring an early inflammatory response that surfaces only at a later time point in WT spinal cord (Figure 3A-B). Other gene expression changes unique to the G93A-SOD1 animal are detected in two later time points within the experimental period. Following the 4-hour time point, the G93A-SOD1 spinal cord displays characteristic apoptotic signals, like the activation of genes regulating cytochrome C release from the mitochondria. Cytochrome C release is known to occur when the inner mitochondrial membrane becomes excessively permeable to ions, leading to energy failure and apoptosis [31,12]. The inflammatory milieu and the activation of cell-death signals in the spinal cord at this stage of the animal recovery may reduce the tissue repair potential contributing to the observed poor functional recovery observed in the G93A-SOD1 rat.

The post-injury molecular profile of the G93A-SOD1 spinal cord does not show the down-regulation of a large number of genes 7 days from compression SCI demonstrated in WT rats compared to naïve controls (Figures 2A-B; 3D). For example, those genes that control cholesterol and isoprenoid biosynthesis remain up-regulated in the G93A-SOD1 spinal cord at this time-point after compression injury. Isoprenoids are biological precursors of carotenes and of coenzyme-Q, retinol and tocopherol (vitamin E), compounds known to modulate mitochondrial activity and oxidative stress [32,33]. A previous report has documented a state of systemic dyslipidemia in individuals with ALS and this state of altered lipid homeostasis has been linked to a potential neuroprotective effect [34]. The presence of a SOD1 gene mutation or of a genetic predisposition to develop ALS may be associated to a state of dysregulation of molecular pathways of cholesterol and isoprenoid biosynthesis, both during the natural development of the disease and in response to a stressful situation like a mechanical injury.

Also down-regulated in WT spinal cord at the 7-day post-injury time point are many genes that like Hcn2 control cell-cell signalling and ion/neurotransmitter transport (GO:0019226; GO:0006836, Figures 3D and 6). Previous experimental observations in animal models of ALS and brain ischemia have reported increased neuronal excitability in affected tissues, generated by the up-regulation of genes capable of modulating ion currents [3,13]. Hence, the acquisition of a state of tissue hyperexcitability may represent a distinguishing feature of SOD1-induced ALS, both during the natural development of the disease and also in a pre-symptomatic phase as a result of a stressful condition.

At the 7 day time-point, both the WT and the G93A-SOD1 spinal cords up-regulate genes involved in retinol (vitamin A) metabolism including RBP1 and CRABP2, whilst only the G93A-SOD1 spinal cord tissue presents the additional up-regulation of ADH1 and ALDH1 (Figure 4B). A link between the vitamin-A down-stream effects and the pathological changes observed in ALS has already been suggested [35-38]. Early vitamin-A deprivation, for example, causes motor cell loss in rodents [35]. RBP1 and CRABP2 are over-expressed in spinal cord from the end-stage G93A-SOD1 rat, whilst surviving small motor neurons show selective immunostaining for specific retinoid receptors [36-38]. Our observations in the acute post-injury phase may inspire potential neuroprotective therapeutic strategies, since both the alteration of ion current regulation and the activation of retinoid signalling can be pharmacologically modified.

We and others have already reported the post-injury down-regulation and/or accumulation of phosphorylated and non-phosphorylated neurofilaments and of synaptophysins (SYN), the latter appearing to be linked to post-traumatic motor and cognitive deficits in different models of neurotrauma [24,25,6,39]. Given the reported trauma-induced differential regulation of Nfh and SYN expression along the motor cell/axon/synapsis axis, we have evaluated the Nfh and SYN differential regulation in our injury model. At a RNA level, Nfh expression appears to be higher in injured G93A-SOD1 spinal cord compared to injured WT tissue from the 4 hour time point onward (Figure 4A). Post-injury protein expression of Nfh measured by immunohistochemistry is also tendentially higher in the G93A-SOD1 spinal cord at all the time points, with a significant level of differential expression at the 24 hour time-point, which was further confirmed with Western blotting (Figures 4, 5, 6, 7, 8). This observation may indicate that neurofilaments expression is altered in a situation of genetic susceptibility to develop ALS, both under conditions of stress and during the natural development of the disease. Intermediate neurofilaments like peripherin within spheroid-like structures are already known to accumulate in affected tissues from animal models of ALS and to possibly interfere with axonal transport [13]. We did not observe any significant differential regulation or any genotype-specific redistribution of SYN in spinal cord after compression SCI. Failure to detect a post-injury change in SYN expression or re-distribution in our study may be related to the relatively low force of impact or to the fact that changes in synaptic vesicle transportation as a consequence of a mild compression SCI may have not yet occurred in its full scale in the 7-day time period. Another indication that SOD1 gene defects may act on the homeostasis of neurofilaments when nervous tissue is under stress is the up-regulation of Mapb1 and possibly of Mast1 in the post-injury G93A-SOD1 spinal cord, both genes appearing to be down-regulated in WT spinal cord after compression injury (Figure 6). Mapb1 binds to microfilaments linking them with the microtubule system, whilst remaining associated to the plasma membranes. Mapb1 exerts a central role in the process of axonal elongation and branching [40]. The observed selective activation of Map1b in G93A-SOD1 spinal cord could be part of a compensative neuroprotective response that counters the intrinsic SOD1-mediated vulnerability under conditions of stress. This survival program may be mediated by the activation of molecules involved in the maintenance of the cytoskeletal integrity as well as by the production of cholesterol and isoprenoids derivates as reported above.

Microarray studies in spinal cord have shown that sham operation alone can induce gene expression changes similar to those caused by mild injury, but only in the first few hours after trauma [8,22]. Our study confirms that simple laminectomy triggers the transcriptional regulation of a wide range of gene categories in both the WT and the G93A-SOD1 spinal cords. However, gene categories involved in transcription, differentiation, ion homeostasis, apoptosis, organisation of mitochondrial function and interleukin-6 release become activated only in the G93A-SOD1 spinal cord at 4 hours from laminectomy. It is clear from our experiments that both laminectomy and mild spinal cord injury provoke a mixture of pro-survival and pro-apoptotic expression changes, the latter being dominated by mitochondrial cell-death signals and interleukin-6-mediated inflammatory changes.

In this study, we have attempted to correlate the poor functional recovery observed in the G93A-SOD1 rats during the acute post-injury phase with changes at the level of individual cell types in the affected spinal cord. We have shown only a trend towards an increase in microglia, macrophages and astrocytes in a cord segment caudal to the injury epicenter in the G93A-SOD1 spinal cord compared to WT littermates under the same experimental conditions, but no significant changes in the number of these cells between the two groups. This observation is in line with a recent study that has analysed the effects of a longitudinal stab injury of the lumbar spinal cord region of the same pre-symptomatic animal model of ALS utilised in our study [41]. In this experimental paradigm of more invasive spinal cord injury, the level of host glial activation and the motor cell numbers at the site of the lesion in a 2-week post-injury period were not significantly different in the injured G93A-SOD1 rats compared to the injured WT littermates [41]. Our study demonstrates that the size of the anterior horns motor cells in the G93A-SOD1 animals after compression SCI is significantly reduced when compared to the WT motor cell population. Previous studies on animal models of ALS have also shown a loss of the largest spinal motor neurons with the disease progression [42]. Various attempts to clarify whether the late stage loss of large motor neurones is due to a higher vulnerability of these neurones, which may die or atrophy earlier than the small ones or whether motor cells never reach their maximal size before disease onset have not come to any conclusion [43]. It is also not known whether the loss or the atrophy of large motor neurons is the main determinant of the functional decline and ultimately of the loss of motor units observed in the overt phase of the disease. Clearly, the same type of uncertainties may apply to the interpretation of our results. The presence of smaller motor neurones in the G93A-SOD1 spinal cord one week after compression injury may play a part in the poor locomotor performances observed in the G93A-SOD1 rats compared to their WT littermates under to the same experimental conditions. The overall molecular changes we have observed in the G93-SOD1 spinal cord in the post-injury phase may also be responsible of the poor functional recovery described in the G93-SOD1 rats.

Similarly to a number of published investigations, our study has used WT littermates as control for the human G93A-SOD1 rats, in order to ensure the maximum level of genetic homogeneity between the groups of animals. This choice is of particular importance when studying changes at a genetic level [6,36,41,44-48]. Previous studies have shown that the human G93A-SOD1 transgene does not affect the endogenous rat SOD1 protein levels [49]. However, it has also been shown that the total SOD1 activity in the G93A-SOD1 rats is increased to 200-300% of the control level, as the result of the combined endogenous SOD1 activity of the rat and of the added mutant G93A-SOD1 activity [49]. This observation is clearly important in the interpretation of our results. In future studies, it will be useful to include rats with elevated non-mutant SOD1 activity as an extra control group, in order to normalise whatever experimental approach to comparable levels of SOD1 activity [50].

## Conclusions

Our study demonstrates a higher level of functional impairment in the pre-symptomatic G93A-SOD1 rats compared to their control WT littermates after mechanical injury of the spinal cord, in the acute 1-week post-injury phase. Comparative gene ontological analysis indicates that the molecular response to a mild compression SCI or to a simple surgical stress in pre-symptomatic G93A-SOD1 rats genetically-modified to develop ALS is distinctively different to that seen in control WT littermates. The response to SCI in the G93A-SOD1 spinal cord seems to replicate some of the most crucial gene expression changes described in the affected tissues during the natural development of ALS and to demonstrate the same changes in the morphology of the motor cell population observed in spinal cord from individuals and animal models of the disease in an advanced stage. The sustained recovery after injury observed in WT rats may be driven, at a molecular level, by a lower level of pro-apoptotic activity coupled with the significant over-expression of factors involved in transcription, angiogenesis, lipid transport and cell adhesion. The results presented here contribute to the understanding of how the genetic background affects the ability of nervous tissues to withstand a mechanical trauma, showing how a G93A-SOD1 gene mutation creates a milieu of gene expression changes which has an unfavourable effect on functional recovery following a mild compression injury to the spinal cord.

## Methods

### Animals

A breeding project was initiated at Taconic USA (Taconic Inc., US), using 5 transgenic Sprague Dawley rats expressing the G93A SOD1 gene mutation and 5 wild-type (WT) littermates as breeding pairs, as previously reported [6]. The G93A-SOD1 breeders were replaced on a monthly basis before any sign of disease onset. An average of 6 pups for each breeding pair were obtained and stored in separate cages. Only female pups were retained for further analysis and tail samples were taken for genotyping. Only female rats have been used to ensure consistence in rat gender with other studies of spinal cord injury (SCI) [6,19]. Since SCI requires manual emptying of bladder to prevent urinary tract infections, we have chosen female animals because their bladders are relatively easier to empty compared to male bladders. A total of 40 heterozygous rats carrying the G93A-SOD1 gene mutation and 40 Wild type (WT) littermates have been shipped to our laboratories at the age of 6 weeks and housed in specific pathogen-free animal facilities at a room temperature of 21°C (under a 12 h light-dark cycle). All animal procedures were conducted according to the Animals (Scientific Procedures) Act 1986 as approved by the United Kingdom Home Office. The G93A-SOD1 transgenic rat model of ALS is known to develop initial signs of motor impairment with either hindlimb or forelimb distribution, at approximately 17 weeks of age, and to progress to end-stage disease in approximately 1 or 2 weeks from disease onset [6]. We have chosen the G93A-SOD1 rat rather than the more widely used G93A-SOD1 mouse because of the need to operate with an animal more suitable to well-established experimental procedures for spinal cord injury.

### Laminectomy and spinal cord compression

Compression SCI was performed on 27 pre-symptomatic G93A-SOD1 transgenic female rats at 10 weeks of age, an early pre-symptomatic stage for these rodent models of ALS. Compression SCI was also performed on 27 WT female age-matched littermates for reference as previously reported [6]. 10 WT and 10 G93A-SOD1 female rats were subjected only to laminectomy, at 10 weeks of age. Surgical procedures were performed as previously described [29,6,19,30]. Following anaesthesia with a mixture of isoflurane (2.5%), oxygen and nitrous oxide (1:1 ratio) at a flow rate of 750--1000 mL min, the skin and muscle surrounding the vertebral column (T10-L1) were incised and a laminectomy was performed at T12 level without damaging the dura. The compression injury was performed by statically applying a 35g weight for 5 min, on a platform (area 2 mm × 5 mm), resting on the dura of the exposed spinal segment. Manual bladder expression after surgery was performed twice daily. As previously reported [6], rats were sacrificed by asphyxiation with carbon dioxide after surgery at the following time points: 5 WT and 5 G93A-SOD1 rats at 30 minutes after compression SCI, 5 WT and 5 G93A-SOD1 rats at 4 hours after compression SCI, 4 WT and 4 G93A-SOD1 rats at 24 hours after compression SCI, 5 WT and 5 G93A-SOD1 rats at 7 days after compression SCI, 3 WT and 3 G93A-SOD1 rats at 30 minutes after laminectomy, 3 WT and 3 G93A-SOD1 rats at 4 hours after laminectomy. In addition, 5 naïve G93A-SOD1 rats and 5 naïve WT littermates were also sacrificed at 10 weeks of age. Naïve spinal cord tissues from WT and G93A-SOD1 animals were used as controls in the differential gene expression analysis described below. Spinal cord samples including the injury site and the adjacent segments (5 mm rostral and caudal to the lesion epicenter), were dissected after sacrifice, submerged into 2-methylbutane and subsequently frozen in liquid nitrogen.

### Locomotor analysis after compression SCI

Following compression SCI, the level of functional recovery in G93A-SOD1 and WT female rats was evaluated initially at 4 hours, and then daily for a week using the BBB locomotor scoring system [20,51,52]. The evaluation of the locomotor function in 24 10-week old WT rats after mild compression SCI in a 7-day post-injury time window has previously been reported [6]. The BBB scores for each animal was determined at the chosen time points by two independent blinded observers and the mean BBB score per group was calculated. All G93A-SOD1 and WT rats have been independently scored using the BBB locomotor functional analysis prior to compression SCI in order to demonstrate similar baseline performances.

### Bead-array gene expression analysis

Large-scale gene expression analysis of the spinal cord samples harvested from the 54 animals after mild compression SCI (epicenters of compression) or laminectomy was performed using the Illumina ratRef-12 v1.0 expression Beadchip (Illumina, San Diego, USA; GEO GPL6101). This Bead-chip contains 12 genome-scale gene expression microarrays (22,523 probes per array). RNA was extracted from the spinal cord samples obtained from injured and uninjured rats, and from sham operated animals subjected to laminectomy, using the SV total RNA isolation system (Promega, UK). RNA quantification was performed using a Nanodrop ND-1000 spectrophotometer and quality was checked using the Agilent bioanalyser system (Agilent). For the purpose of our gene expression analysis, we have pooled the RNA samples obtained from the injury epicenters that have been dissected from rats of the same genetic type and sacrificed at the same post-injury time-point. We have obtained a total of 14 RNA-pools (4 pools containing RNAs from G93A-SOD1 cords extracted at the 30 min.(n:5), 4 hours (n:5), 24 hours (n:4) and 7 days (n:5) time points after compression SCI respectively, 2 pools containing RNAs from G93A-SOD1 spinal cords extracted at the 30 min. (n:4) and the 4 hours (n:4) time points after laminectomy, 2 pools containing RNAs from WT spinal cords extracted at the 30 min. (n:4) and at the 4 hours (n:4) time points after laminectomy, 4 pools containing RNAs from WT cords extracted at the 30 min (n:5), 4 hours (n:5), 24 hours (n:4) and 7 days (n:5) time points after compression SCI respectively; finally 1 pool containing G93A-SOD1 (n:5) and 1 pool containing WT (n:5) spinal cords extracted from 10 week-old naive animals (used as references in the gene expression analysis) of which we have tested the gene expression profile. The pool of RNA samples from G93A-SOD1 spinal cords harvested at 30 minutes from compression injury was also used for a technical replica experiment.

cRNA labeling was performed with 750 ng of pooled RNA, using the Ambion Total Prep kit. cRNA purity and labelling was checked using the Nanodrop and Agilent bioanalyser. 500 ng from each of the 14 RNA pools were hybridised simultaneously to 16 arrays (including 2 arrays for the technical replica experiment) contained in 2 RatRef-12 Expression BeadChips, as per Illumina protocol (Illumina, San Diego, USA).

#### Differential gene expression and correlation analysis

the Bead-chip output files were analyzed using the BeadStudio-3 Software (Illumina, San Diego, USA) as previously reported [6]. The expression level of each probe was defined by a value of intensity and by a p-value detection, which measures the probability of signal recovery without specific probe target hybridization. Genes differentially expressed at the selected time-points after compression SCI and laminectomy were identified by comparing G93A-SOD1 and WT spinal cord tissues with genetically-matched naïve tissues. We have also performed a differential gene expression analysis with a direct comparison of G93A-SOD1 and WT tissues at the selected time-points after injury. This approach allows the identification of gene candidates presenting different expression behaviours in the two tissues in study, but does not indicate the nature of the expression change in each spinal cord type compared to genetically-matched un-injured (naïve) tissue. Therefore, genetically-matched naïve tissues were used as a reference for data interpretation. The gene expression changes identified in spinal cord from the naive (10-week old) G93A-SOD1 rats (using the spinal cord tissue from age-matched WT littermates as reference), have been taken into account for the interpretation of the gene expression results obtained from injured spinal cord samples and from spinal cords dissected from animals subjected to laminectomy.

Stringent selection criteria (Bead Studio-3), including a rank invariant normalization algorithm, an Illumina Custom error model (to compute non-specific cross-hybridization and the effects of variation arising from non-biological factors) and a false discovery rate (FDR) were used in the differential gene expression analysis as previously described [6]. To evaluate the reproducibility of our results, we have performed a correlation test (BeadStudio scatter plot analysis) between the gene expression profiles generated from the G93A-SOD1 and the WT spinal cord tissues, with those obtained from naive control tissues of the corresponding genetic type. We have also extracted from the array data, the intensity values of detection at the various experimental time points after compression SCI for Nfh and for other gene candidates involved in retinoid signaling (ReS).

### Ontology analysis of the gene expression data

We have used *High-Throughput GoMiner *as previously reported [6,21] for an ontological analysis of the gene expression changes in spinal cord tissue from WT and G93A-SOD1 animals after compression injury and laminectomy. *High-Throughput GoMiner *provides an integrated biological interpretation of multiple gene expression datasets. The program identifies those gene categories within the Gene Ontology (GO) database which present a significant level of enrichment of the differentially regulated genes under investigation. We have submitted to *High-Throughput GoMiner *9 text files reporting "changed genes" found to be differentially regulated after compression SCI in G93A-SOD1 and in WT spinal cord pools extracted at 30 minutes, 4 hours, 24 hours and 7 days from injury (tissues from genetically-matched naïve rats sacrificed at the same time points were used as references) and in the 10-week old G93A-SOD1 uninjured spinal cord pool compared to a WT naïve spinal cord pool from age-matched littermates. A set of four text files containing "changed genes" identified in the G93A-SOD1 and in the WT spinal cord pools at 30 minutes and 4 hours after laminectomy were submitted separately, along with a list of all the probes contained in the Illumina rat Beadchip. As previously reported, a flat +1 or -1 binary code was used to express up-regulation or down-regulation of the genes included in the 13 text files [6]. GO categories were identified and sorted according to a false discovery rate (FDR; threshold of 0.05) and to the level of enrichment of "changed genes". A multiple comparisons correction was used to eliminate GO categories that appeared significantly represented simply by chance. The FDR value for a specific GO category identified in a text file was automatically computed for all the remaining text files, allowing an estimation of the relative importance of each gene category across all time points and experimental groups. Visual integration of the results was obtained using clustered image maps and an Excel drawing platform and the results have been displayed as heat maps as previously reported [6].

### cDNA synthesis and real time RT-PCR

RNA from the epicenters of injury in the thoracic spinal cord samples obtained from rats of the same genetic type sacrificed at the same time point from compression injury were pooled together (5 samples for each pool) and used for cDNA synthesis using the ImProm-II Reverse Transcription System (Promega, Madison, Wisconsin). 2.5 μg of total RNA from each pool was treated with RNase-free DNase and then heated to 75°C for 10 minutes for DNase inactivation and RNA denaturation. Reverse-transcription was carried out with 1 μl random primers (150 ng/μl) and with 1 μg of RNA. A mixture of 4 μl 5 × reaction buffer, 2 μl of 25 mM MgCl_2_, 1 μl of 10 mM dNTP mix, 0.5 μl recombinant RNasin ribonuclease inhibitor (40 U/μl) and 1 μl ImProm-II™ Reverse Transcriptase (Promega, Madison, Wisconsin) was added to each sample and double distilled H_2_O up to a total of 15 μl. The samples were incubated at 25°C for 5 minutes, then at 55°C for 60 minutes and finally at 70°C for 15 minutes. Negative control had no ImProm-II™ Reverse Transcriptase.

#### Real time RT-PCR

Real time RT-PCR was performed with SYBR Green (ABgene, Epsom, UK), using a Rotor-Gene 3000 system (Corbett Research, Sydney, Australia). Primers (Invitrogen, Paisley, UK) were designed and used in the amplification process for the following genes: the neurofilament heavy chain (Nfh F: AGA GGC AGA AGA GGG AGG AG - Nfh R: TGA CCT CAG CTG GTG ACT TG); the microtubule-associated protein 1B (Map1b 1 F: CCT GCC AAA GAA CTT GAA GC - Map1b 1 R: CCT TTG CTG ACT TCC GTC TC); S100A8 protein (S100A8 1 F: GGC AAC TGA ACT GGA GAA GG - S100A8 1 R: ACC CTT ATC ACC AAC GCA AG); nucleotide-gated potassium channel 2 (Hcn2 F: CTG CGT GAG GAG ATT GTG AA - Hcn2 R: TTT GAG CTT TGT CAG CAT GG); the tissue inhibitor of metalloproteinase 2 (Timp2 1 F: CAA GTT CTT TGC CTG CAT CA - Timp2 1 R: TCC AGG AAG GGA TGT CAA AG). Amplification conditions were as follows: 95°C for 15 min, followed by 40 cycles of 95°C for 10 s; 60°C for 15 s; 72°C for 20 s. Each real time PCR experiment was performed in triplicate, using cDNA templates originated from 2.5 μg of RNA. The expression of the test genes was evaluated following normalization to the level of ribosomal 18 S contained in each sample. Due to the 18 S abundance in the tissues, the 18 S cDNAs were diluted 100-fold before RNA measurement. A standard curve was generated by real-time RT-PCR analysis from triplicates of five ten-fold dilutions of cDNA generated from 1 ng of spleen RNA. Real time PCR runs showing mRNA expression signals for the negative control samples were discarded and new cDNA was generated for a re-run.

### Immunohistochemistry

The protein expression of the neurofilament heavy chain (Nfh) and of synaptophysin (SYN) in spinal cord after compression SCI has been studied by immunohistochemistry. Spinal cord samples immediately caudal to the site of compression SCI were harvested from 3 WT and 3 G93A-SOD1 rats at 30 min, 4 h, 24 h and 7 days after compression SCI and from naïve 10-week old rats, for immunostaining. Tissue samples were frozen in liquid nitrogen and stored at -80°C. Fresh frozen tissues were mounted on tissue holders and 15 μm sections were cut on a cryostat. Slides with fresh frozen tissue were post-fixed with 100% ethanol for 10 minutes, followed by 3 washes for 10 minutes in phosphate buffered saline (PBS). Endogenous peroxidases were inhibited by treating slides with 0.3% H_2_O_2 _for 10 minutes. After 3 × PBS washes for 10 minutes, non-specific antibody sites were blocked with 10% donkey serum in 0.3% triton/0.1% sodium azide PBS solution for 2 h. Primary anti-mouse antibodies for Nfh (clone N52, 1:20,000, Sigma) and SYN (1:5,000, Sigma) diluted in 0.3% triton/0.1% sodium azide and PBS solution were applied for 24 h followed by biotinylated secondary anti-mouse (for Nfh) and anti-rabbit (for SYN) antibodies at dilution 1:800 in 0.3% triton/0.1% sodium azide PBS solution for 1 h. Vectastain Elite ABC Kit (Vector laboratories) was subsequently added for 30 minutes to form an avidin-biotin complex. The reaction was visualised after incubation with 3, 3'-diaminobenzidine (DAB, Sigma) and sections were mounted in xylene-based DPX mounting medium. The Nfh antibody (clone N52) has been previously used in our lab and published by other authors. It has been extensively characterised by Western blot analysis, tested for cross-reaction with other neurofilaments, and it has become an established marker for myelinated DRG neurons [53,51,52,27]. The synaptophysin antibody (Sigma S5768; mouse synaptosome preparation from rat retina -1:500 dilution) has also been extensively tested and its immunoreactivity has been quantified using the same method previously reported [54].

Histopathological assessment of the WT and G93A-SOD1 rat spinal cord after mild compression injury using luxol fast blue staining and inflammatory markers was performed as previously described [26-29,54-56]. We have used frozen 20 μm transverse spinal cord sections caudal to the compression site from animals sacrificed 7 days after compression injury. Sections were dehydrated in a graded ethanol series (5 min each) and then incubated overnight in 0.1% luxol fast blue solution at 37°C. Differentiation of slides was carried out with 0.05% lithium carbonate to distinguish white and gray matter staining and then dehydrated in a graded ethanol series. Slides were dipped in xylene and mounted in DPX mounting medium. Slides with fresh frozen tissue for immunohistochemistry were post-fixed with 4% paraformaldehyde for 1 h, followed by 3 washes for 5 minutes in PBS. Immunofluorescent staining with mouse anti- ED1 (1:400, Chemicon), mouse anti-OX42 (1:200, Serotec) and rabbit anti-GFAP (1:1000, Dako) was carried out overnight at room temperature. After 3 × PBS washes for 5 minutes, secondary donkey anti-mouse AlexaFluor 488 or goat anti-rabbit AlexaFluor 568 (1:1000, Invitrogen) was applied for 3 h at room temperature. Nissl staining of motor neurones was carried out using Neurotrace fluorescent Nissl stain (1:100, Invitrogen) according to manufacturer's instruction. After a further 3 × PBS washes for 5 minutes, sections were mounted in Vectashield mounting medium containing with or without DAPI (Vector Labs).

A summary of the specifications of the antibodies used for the immunohistochemical analyses is presented in Table 1.

**Table 1:** Main characteristics of the antibodies used for the experiments detailed in the manuscript.

	Manufacturer	Product & Lot No.	Host species	Immunogen	Dilution used
**Primary antibodies**					
Neurofilament (clone N52)	Sigma	N0142 & 65K4804	mouse	C-terminal segment of enzymatically dephosphorylated pig neurofilament 200	1:20,000 using immunoperoxidase staining 1:1500 using Western blotting
Synaptophysin (clone SVP-38)	Sigma	S5768 & 90K4844	mouse	Rat retina synaptosome	1:5000 using immunoperoxidase staining
Rat CD68- macrophages/monocytes (clone ED-1)	Chemicon	MAB1435 & 0606032963	mouse	A single chain glycoprotein of 90-100 KDa that is expressed on the lysosomal membrane of myeloid cells	1:400 using immunofluorescent staining
Rat CD11b; RPE (clone OX-42)	Serotec	MCA275EL & 0105	mouse	Rat peritoneal macrophages	1:200 using immunofluorescent staining
Glial fibrillary acid protein (clone SVP-38)	Dako	Z0334 & 00045904	rabbit	Cow spinal cord	1:1000 using Immunofluorescent staining
β-actin (clone AC-15)	Sigma	A1978 & 118K4827	mouse	Fusion of mouse myeloma cells and splenocytes immunized with a synthetic b-cytoplasmic actin N-terminal peptide	1:5000 using Western blotting
NeuroTrace (fluorescent Nissl stain)	Molecular Probes	N21480 & 4971-21	-	-	1:100 according to manufacturer's instruction
**Secondary antibodies**					
Alexa Fluor 488	Invitrogen	A21202 & 536050	donkey	Anti-mouse IgG (H+L)	1:1000 using immunofluorescent staining
Alexa Fluor 568	Invitrogen	A10042 & 538952	donkey	Anti-rabbit IgG (H+L)	1:1000 using immunofluorescent staining
Alexa Fluor 680	Invitrogen	A21057 & 65E2-1	goat	Anti-mouse IgG (H+L)	1:15,000 using Western blotting

### Western blotting

A spinal cord segment rostral to the compression site was freshly collected from 3 G93A-SOD1 and from 3 WT age-matched rats 24 hours from compression SCI and stored at -80°C until processed. The spinal cord tissues were processed for Western blot as previously described [57]. Briefly, spinal cord tissues were homogenized in 1 ml of ice-cold lysis buffer (20 mM HEPES pH 7.4, 100 nM NaCl, 100 mM NaF, 1 mM Na_3_VO_4_, 5 mM EDTA, 1% Nonidet P-40 and 1 × protease inhibitor cocktail; Roche). The lysates were rotated for 2 h at 4°C then centrifuged at 13,500 g for 15 min at 4°C. Total protein concentration was determined for the collected supernatant using a bicinchoninic acid protein assay kit (Pierce). Fifteen micrograms of total protein were electrophoresed on 12% acrylamide gel before transfer onto Hybond P membranes (Amersham) and incubated overnight at 4°C with mouse anti-NF200 (clone N52, 1:1,500) and mouse anti-β-actin (1:5,000). Although both antibodies were raised in mouse, the difference in molecular size (NF200 is 200 KDa and β-actin is 42 KDa) allows for simultaneous probing on the same blot. Visualisation was performed using the secondary antibody goat anti-mouse IRdye-680CW (LI-COR Biosciences). Fluorescent blot was imaged on the Odyssey Infrared Imaging System (LI-COR Biosciences). The bands were quantified using AxioVision 4.6 program and the results were expressed as a ratio with its corresponding internal control β-actin.

#### Histological analysis

The intensity of Nfh staining in motor neurons (10 -- 15 per section) was measured at 20 × magnification using Scion Image (release alpha 4.0.3.2). The average intensity of Nfh staining (gray level) of motor neurons was measured and the average background staining intensity was subtracted. Analysis of SYN-immunoreactive synaptic boutons was performed at 40× magnification. The average intensity (gray levels) of SYN-stained nerve terminals and subtracted the average staining intensity of the background was determined. The analysis of ED1, OX42 and GFAP immunostaining was determined similarly to previously described [28,54]. Spinal cord sections were captured with the same intensity and settings using the Zeiss Axioplan 2 fluorescence microscope. The number of positively stained ED1, OX42 and GFAP cells was determined at 20 × magnification in a 1 × 10^4 ^μm^2 ^box place over the ventral horn using the Axiovision 4.6 program.

To determine the amount of spared white matter caudal to the injury, the area of white matter positively stained with luxol fast blue was measured using the Axiovision 4.6 program. All analyses for immunohistochemical staining were performed in the ventral spinal cord of 6-8 non-adjacent sections of a spinal cord segment caudal to the epicenter of injury from 3 WT and 3 G93A-SOD1 animals.

### Motor neurone analysis: numbers and cell size

The count of motor neurones and the analysis of the motor neurones cell size was carried out as previously described [57]. Cell area of Nissl labelled motor neurones with visible DAPI positive nuclei in the spinal cord ventral horn was acquired using the AxioVision V4.6 program. Size and frequency distributions of motor neurones were determined for each rat and a mean distribution calculated for both G93A-SOD1 and WT rats. At least six spinal cord sections were analysed and quantified per rat (*n *= 3-4 per group). Statistical analysis was carried out using a two-sample Kolmogorov-Smirnov test, performed against a significant threshold of 0.05 to correct for multiple testing.

### Statistical analysis

The data were presented as means and standard errors of the means (SEM). The behavioural data were analysed with two-way ANOVA followed by Tukey's *post-hoc *test. Histological and Western blot data were analysed with Student's t-test. P < 0.05 was considered statistically significant.

## Authors' contributions

NJ has carried out all the surgical procedures of sham operation and spinal cord compression. She has performed all the functional analyses and processed all the tissue samples for the Bead-Array analysis and RT-PCR described in the study. PKY and NJ have carried out all the histological, immunohistochemical work, along with cell count and analysis of the images. AMT and JVP participated in the design of the study and have supervised the direction of the experiments and the statistical analyses. AM has performed all the gene expression data analyses and interpretation using the bioinformatic tools reported in the manuscript. AM has conceived the study, and participated in its design and coordination. All authors have read and approved the final manuscript.
